# The long noncoding RNA SUMO1P3 as urinary biomarker for monitoring bladder cancer progression

**DOI:** 10.3389/fonc.2024.1325157

**Published:** 2024-05-23

**Authors:** Silvia Galbiati, Arianna Bettiga, Giorgia Colciago, Chiara Senti, Francesco Trevisani, Giulia Villa, Ilaria Marzinotto, Michele Ghidini, Rodolfo Passalacqua, Francesco Montorsi, Andrea Salonia, Riccardo Vago

**Affiliations:** ^1^Complications of Diabetes Unit, Diabetes Research Institute, IRCCS San Raffaele Scientific Institute, Milan, Italy; ^2^Urological Research Institute, Division of Experimental Oncology, IRCCS San Raffaele Scientific Institute, Milan, Italy; ^3^Oncology Unit, ASST of Cremona, Hospital of Cremona, Cremona, Italy; ^4^Center for Nursing Research and Innovation, Vita-Salute San Raffaele University, Milan, Italy; ^5^Beta Cell Biology Unit, Diabetes Research Institute, IRCCS San Raffaele Scientific Institute, Milan, Italy; ^6^Faculty of Medicine and Surgery, Università Vita-Salute San Raffaele, Milan, Italy

**Keywords:** SUMO1P3, bladder cancer progression, urinary biomarker, liquid biopsy, long non-coding RNA

## Abstract

**Introduction:**

Urothelial Bladder Cancer (BC) is the ninth most common cancer worldwide. It is classified into Non Muscle Invasive (NMIBC) and Muscle Invasive Bladder Cancer (MIBC), which are characterized by frequent recurrences and progression rate, respectively. The diagnosis and monitoring are obtained through invasive methods as cystoscopy and post-surgery biopsies. Thus, a panel of biomarkers able to discriminate BC based on grading or staging represents a significant step forward in the patients’ workup. In this perspective, long non-coding RNAs (lncRNAs) are emerged as reliable candidates as potential biomarker given their specific and regulated expression. In the present work we propose two lncRNAs, the Small Ubiquitin Modifier 1 pseudogene 3 (SUMO1P3), a poorly characterized pseudogene, and the Urothelial Carcinoma Associated 1 (UCA1) as candidates to monitor the BC progression.

**Methods:**

This study was a retrospective trial enrolling NMIBC and MIBC patients undergoing surgical intervention: the expression of the lncRNA SUMO1P3 and UCA1 was evaluated in urine from 113 subjects (cases and controls). The receiver operating characteristic curve analysis was used to evaluate the performance of single or combined biomarkers in discriminating cases from controls.

**Results:**

SUMO1P3 and UCA1 expression in urine was able to significantly discriminate low grade NMIBC, healthy control and benign prostatic hyperplasia subjects versus high grade NMIBC and MIBC patients. We also demonstrated that miR-320a, which binds SUMO1P3, was reduced in high grade NMIBC and MIBC patients and the SUMO1P3/miR-320a ratio was used to differentiate cases versus controls, showing a statistically significant power. Finally, we provided an automated method of RNA extraction coupled to ddPCR analysis in a perspective of clinical application.

**Discussion:**

We have shown that the lncRNA SUMO1P3 is increased in urine from patients with high grade NMIBC and MIBC and that it is likely to be good candidate to predict bladder cancer progression if used alone or in combination with UCA1 or with miRNA320a.

## Background

Bladder cancer is the most common malignancy of the urinary tract (1) and is widespread worldwide, especially in Europe and North America (2). Most patients (about 75%) are diagnosed with non-muscle-invasive bladder cancer (NMIBC), which is characterized by a high recurrence rate and progression to muscle-invasive bladder cancer (MIBC). The prognosis of bladder cancer remains strictly related to the aggressiveness at the time of diagnosis with MIBC, which spreads throughout the surrounding tissues and to distant sites, featuring a low 5-year overall survival (around 30%) (3). To date, the diagnostic procedure for bladder cancer mainly relies on the combination of cystoscopy and cytology, the first being invasive, which limits patient compliance and mass screening, the latter from modest specificity (4). To overcome these limitations, a few biomarkers have been proposed in the context of liquid biopsy; however, to date, no one has entered clinical practice.

An increasing number of studies have demonstrated that cancer-related long noncoding RNAs (lncRNAs) aberrant expression and mutations are strongly associated with tumorigenesis, metastasis, and tumor stage (5, 6). To date, multiple lncRNAs have been found to support or inhibit cell proliferation and their dysregulation contributes to cancer cell growth. For instance, Urothelial Cancer Associated 1 (UCA1) is a well-documented lncRNA in bladder cancer that promotes tumorigenic potential and drug resistance (7), and H19 RNA levels are found to be much higher in both primary and metastatic tumor than in normal tissues (8). The recently described small ubiquitin-like modifier 1 pseudogene 3 (SUMO1P3) is a pseudogene-expressed lncRNA previously identified in gastric cancer tissues (9) and has been reported to be involved in other tumors, such as colon (10), breast (11), and liver cancers (12). It promotes tumor growth, metastasis, and angiogenesis (10), and SUMO1P3-knockdown inhibits cell proliferation and migration while inducing apoptosis (13).

LncRNAs are versatile regulators of gene expression, which interact with DNA, RNA, and proteins and exert their function through a variety of mechanisms by acting as scaffolds, decoys, and recruiters of genetic modifiers (14). One of them is their interaction with miRNAs, whose roles in gene regulation and cell function have been elucidated in numerous cancers (15). MiR-507 has been shown to directly bind to UCA1, and a negative correlation has been shown between them. FOXM1, a target of miR-507, can be downregulated by either miR-507 overexpression or UCA1 depletion, providing evidence of a regulatory network in tumorigenesis of melanoma (16). MiR-200a binds to H19 and inhibits its expression, thereby decreasing colorectal cancer cell proliferation. This competitive binding regulates *β*-catenin expression and activity and promotes cell proliferation (17). The tumor-promoting effects of SUMO1P3 in breast cancer are at least partly mediated by negative regulation of miR-320a (11).

Because of their roles in cancer onset and development, lncRNAs have attracted interest as therapeutic targets (18) and biomarkers (19). The poor prognosis of various types of cancers is at least partly due to the lack of timely early diagnosis. Therefore, a major unmet clinical need in bladder cancer management includes noninvasive methods for disease surveillance, and the identification of robust biomarkers to predict disease progression can play a pivotal role in improving patients’ lives and management.

In the present work, we show that two lncRNAs, Urothelial Carcinoma Associated 1 (UCA1) and Small Ubiquitin Modifier 1 pseudogene 3 (SUMO1P3), were increased in the urine of bladder cancer patients affected by high-grade and muscle-invasive tumors with respect to superficial, low-grade tumors, healthy controls, and men with benign prostate hyperplasia, indicating that UCA1 and SUMO1P3 are good candidates for monitoring tumor progression.

## Methods

### Patients’ enrolment and urine collection

Caucasian patients diagnosed with urothelial carcinoma of the bladder or benign prostatic hyperplasia (BPH) were recruited. Patients with concomitant or previous diagnoses of prostate, renal, or upper excretory tract cancer, urinary tract infections, or kidney failure were excluded. Control urine samples were obtained from healthy volunteers without any current or previous diagnosis of cancer or other pathologies in relation to the urinary tract. All studies carried out on patient samples were approved by the Institutional Ethical Committee of the San Raffaele Hospital, and informed consent was obtained. Urine samples were collected before the surgical intervention and processed soon after. The samples were centrifuged at 300×*g* for 5 min, aliquoted, and stored at −80°C until use.

### RNA extraction

RNA was isolated from 2 ml of urine using the QIAamp Circulating Nucleic Acid Kit (Qiagen GmbH, Hilden, Germany), following the manufacturer’s instructions. Isolated RNA was eluted in 30 µl–50 µl of nuclease-free water; concentration and purity were assessed using an ND-1000 spectrophotometer (NanoDrop Technologies, Wilmington, USA), and absorbance at 260 nm/280 nm was used as a reference of purity; when it was not optimal, a further purification step was carried out with the RNeasy Plus Minikit kit (Qiagen Gmbh, Hilden, Germany).

### RNA expression profiling using NanoString

Direct RNA analysis was performed using an nCounter Digital Analyzer (NanoString, Diatech). The nCounter Standard Master kit, which includes all reagents, sample cartridges, and consumables necessary for sample processing, was used. A multiplex nCounter Custom CodeSet (Nanostring, Diatech) was designed to evaluate the 96 long non-coding RNAs selected for the analysis (**Supplementary Table 1**).

### Automatized RNA extraction and processing

Eight ml of urine were centrifuged at 300*g* for 5 min and concentrated to 0.5 ml with Amicon Ultra Centrifugal Filter 10 kDa molecular weight cutoff (Merck, Darmstadt, Germany). RNA was isolated using the Maxwell^®^ RSC miRNA Plasma and Serum Kit (Promega, Madison, WI, USA) following the manufacturer’s instructions. The RNA was eluted in 50 µL of nuclease-free water, and 10 µL was retrotranscribed with a High Capacity cDNA Reverse Transcription Kit (Applied Biosystems) for lncRNA analysis. The TaqMan^®^ Advanced miRNA cDNA Synthesis Kit was used to prepare the cDNA template (starting from 3.7 µL of total RNA), combined with the TaqMan^®^ Advanced miRNA Assays specific for the detection of miR-320a by ddPCR.

### Digital droplet PCR reagents and cycling conditions

SUMO1P3, LOC100506990, UCA1 lncRNA, and miR-320a expression levels were determined by droplet digital PCR (ddPCR) using a QX100 ddPCR platform (Bio-Rad, Hercules, CA, USA). The volume of the PCR mix was 20 µL, including 10 µL of ddPCR™ Supermix for Probes (No dUTP, Hercules, CA, USA), 1 µL of probe (specific for each lncRNA/miRNA provided by Thermo Fisher, UCA1 = Hs01909129_s1, SUMO1p3 = Hs01689249_s1, LOC100506990 = Hs04274800_m1, and hsa-miR-320a—Assay ID: 478594_mir), and 9 µL or 2 µL of cDNA template for the analysis of lncRNAs or miR-320a, respectively.

The droplet emulsion was thermally cycled using a C1000 Touch Thermal Cycler (Bio-Rad). Cycling conditions were 95°C for 5 min, followed by 40 cycles of amplification (94°C for 30 s and 55°C for 1 min), and ending at 98°C for 10 min, according to the manufacturer’s protocol. The concentration of the target was calculated automatically using QuantaSoft™ software version 1.7.4 (Bio-Rad).

### Statistical analysis

All analyses were performed using R software, version 4.1.2 (https://www.R-project.org). Descriptive statistics (mean ± standard deviation or percentage of the total number of subjects) were used to summarize the major demographic and clinical features of the study cohort. One-way ANOVA was performed to compare the means of each lncRNA urinary level across multiple groups, e.g., histologically different bladder cancer patients and controls. Then, Tukey Honest Significant Differences (HSD) *post-hoc* analysis was conducted to assess differences in lncRNA levels between two single groups, with the correction of p-values for the number of comparisons. Statistical significance was set at P <0.05. Receiver operating characteristic (ROC) curve analysis was used to evaluate the performance of single and combined biomarkers in discriminating cases from controls. The area under the ROC curve (AUC) was calculated using the pROC R package.

## Results

### Discovery of candidate lncRNAs in urine from bladder cancer patients

A group of 84 urine samples derived from bladder cancer patients who underwent surgical intervention and healthy controls were used for a retrospective study aimed at identifying lncRNAs linked to tumor progression. The patients were divided into five groups according to disease staging and grading. In addition to healthy controls, patients with Benign Prostatic Hyperplasia (BPH) were included in the study as a non-tumoral disease affecting another organ of the urinary tract, representing an important step to assess the specificity of the analysis. RNA isolated from urine underwent a further purification step due to its low purity to make its quality compatible with the following analysis. Only samples with a concentration higher than 15 ng/ul and with ascertained purity were used for the next analysis; 48 of 84 samples satisfied these parameters and were analyzed using the NanoString platform. The characteristics of the patients in this first cohort are summarized in **Table 1**.

**Table 1:** Data from the first cohort of patients.

pathological grade/stage	NMIBC	NMIBC	MIBC	BPH	Controls
	Ta G1–G2 low grade	T1 G1–G2 high grade	T2–T4 high grade		
	(n = 10)	(n = 7)	(n = 15)	(n = 7)	(n = 9)
Mean age ± SD	58.1 ± 14.5	72.7 ± 6.1	71.6 ± 6.9	67 ± 0.3	52.7 ± 7.3
Gender (%)					
male	8 (80)	7 (100)	14 (93)	7 (100)	6 (67)
female	2 (20)	0 (0)	1 (7)	0 (0)	3 (33)
Carcinoma *in situ* (CIS) (%)					
Yes	1 (10)	3 (43)	2 (13)	0 (0)	0 (0)
No	9 (100)	4 (57)	13 (87)	7 (100)	9 (100)
Node positive (%)					
Yes	0 (0)	0 (0)	6 (40)	0 (0)	0 (0)
No	10 (100)	7 (100)	9 (60)	7 (100)	9 (100)
First episode (%)					
Yes	8 (80)	5 (71)	15 (100)	7 (100)	
No	2 (20)	2 (29)	0 (0)	0 (0)	

NMIBC, Non-Muscle Invasive Bladder Cancer; MIBC, Muscle-Invasive Bladder Cancer; BPH, Benign prostatic hyperplasia.

**Figure 1:**
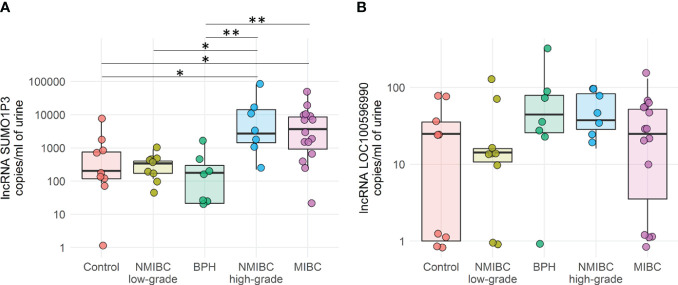
Quantification by ddPCR of SUMO1P3 **(A)** and LOC100596990 **(B)** levels expressed as copies/ml in 48 urine samples derived from bladder cancer patients and healthy controls. The ANOVA test with the Tukey HSD *post hoc* analysis was used to determine significant differences between groups (**p-value <0.01, *p-value <0.05).

A panel of 96 target lncRNAs was used as a probe, some of which have been previously demonstrated to be involved in the development of bladder cancer or other tumors (UCA1, MALAT1, H19, and HOTAIR) (20), while others have not yet been characterized. One or more lncRNA targets were detected in only 16 out of 48 samples, belonging to the high-grade NMIBC, MIBC, and healthy control groups. No data were available at this step from low-grade NMIBC and BPH, likely due to the very low amount of tumoral lncRNA. SUMO1P3 and the uncharacterized LOC100506990 showed significantly higher expression in patients affected with high-grade NMIBC and MIBC (**Supplementary Table 1**). All samples were then reanalyzed by droplet digital PCR (ddPCR), a quantitative and sensitive tool to investigate gene expression, to confirm the obtained results. RNA from the same urine samples was reverse-transcribed to cDNA and then subjected to ddPCR analysis. Both lncRNAs were detectable with SUMO1P3, much more expressed than LOC100506990, which showed a very low signal (**Figure 1**).

SUMO1P3 was significantly more expressed in the urine of patients with MIBC or high-grade NMIBC than healthy controls and BPH patients, and could discriminate between low- versus high-grade NMIBC.

Remarkably, in urine samples derived from patients diagnosed with BPH, SUMO1P3 levels were comparable to those of low-grade NMIBC and healthy controls and were significantly reduced compared to invasive and high-grade tumors. Therefore, these data allowed us to exclude false positives in patients with BPH. The overall data obtained from this first patient cohort demonstrated that SUMO1P3 is a good biomarker for discriminating bladder tumors with aggressive phenotypes from healthy low-grade NMIBC.

The expression of LOC100506990 was much lower than that of SUMO1P3 lncRNA in a cohort of 48 urine samples under these experimental conditions. Moreover, it does not seem to correlate with bladder cancer staging or grading, as the values are homogenous among different groups. For this reason, this has not been investigated further.

### Validation of the SUMO1P3 diagnostic potential

To validate the potential use of SUMO1P3 as a biomarker for the progression of bladder cancer from superficial to more aggressive phenotypes, we defined a validation cohort composed of 65 new patients, the characteristics of which are summarized in **Table 2**. UCA1 was included in the panel because of its documented correlation with different tumors, including bladder cancer (7).

**Table 2:** Data from the validation cohort of patients.

pathological grade/stage	NMIBC	NMIBC	MIBC	T0G0
	Ta G1–G2 low grade	T1 G1–G2 high grade	T2–T4 high grade	
	(n = 17)	(n = 16)	(n = 17)	(n = 15)
Mean age ± SD	59.2 ± 10.4	73.5 ± 11.6	72.1 ± 9.3	65.3 ± 12.2
Gender (%)				
male	14 (82)	14 (87,5)	15 (88)	12 (80)
female	3 (18)	2 (12,5)	2 (12)	3 (20)
Carcinoma *in situ* (CIS) (%)				
Yes	0	1 (6)	3 (18)	0
No	17 (100)	15 (94)	14 (82)	15 (100)
First episode (%)				
Yes	15 (88)	7 (44)	7 (41)	1 (7)
No	2 (12)	9 (56)	10 (59)	14 (93)
Smoking (%)				
Ever	12 (71)	10 (62)	11 (65)	10 (67)
Never	5 (29)	6 (38)	7 (35)	5 (33)

NMIBC, Non-Muscle Invasive Bladder Cancer; MIBC, Muscle-Invasive Bladder Cancer; T0G0, No tumor detected at the histological evaluation.

In this validation cohort, in addition to the low- and high-grade NMIBC and MIBC already present in the first cohort, urine samples from T0G0 patients were considered. People with a T0G0 diagnosis were suspected to be affected by bladder cancer, but histopathological analysis following surgery did not show any detectable tumor. These samples could represent possible false positives and were included in the analysis to test the robustness of the method in the presence of potential confounding factors.

**Figure 2:**
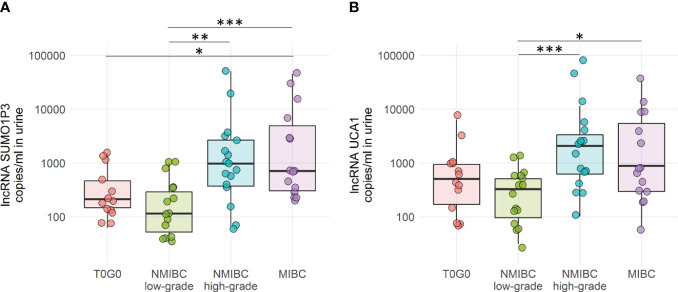
Droplet digital PCR quantification of SUMO1P3 **(A)** and UCA1 **(B)** levels expressed as copies/ml in 65 urine samples derived from bladder cancer patients and controls. The ANOVA test with the Tukey HSD *post-hoc* analysis was used to determine significant differences between groups (***p-value <0.001, **p-value <0.01, *p-value <0.05).

We initially focused on an extraction system to isolate RNA from urine to set up a more efficient process and to automate the process, making it more compatible with clinical procedures. A urine volume of 8 ml of urine was found to be suitable and compatible with the availability of the biobanked sample. Samples were concentrated to 0.5 mL and RNA extraction was performed using the automatic extractor Maxwell^®^ RSC. The RNA obtained was reverse transcribed and analyzed by ddPCR to evaluate the amount of SUMO1P3 and UCA1 in all samples belonging to the validation cohort (**Figure 2**).

**Figure 3:**
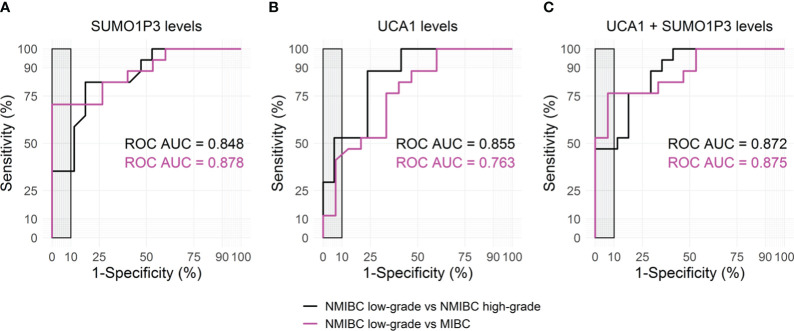
Receiver Operating Characteristic (ROC) curves for SUMO1P3 **(A)**, UCA1 **(B)**, and the combination of SUMO1P3 and UCA1 **(C)** levels, detected in urines from bladder cancer patients, predicting the aggressive tumor phenotypes. ROC curve analyses were performed using the low-grade NMIBC samples as controls and the high-grade NMIBC (black) or the MIBC samples (magenta) as cases. On each graph, the corresponding area under the ROC Curve (AUC) is indicated. The gray rectangle highlights the area in which the specificity of the test is >90%.

Both SUMO1P3 and UCA1 resulted significantly more highly expressed in samples from high-grade NMIBC and MIBC with respect to low-grade NMIBC confirming, in case of SUMO1P3, the previous results obtained from the first cohort. Moreover, SUMO1P3 was significantly more expressed in samples from MIBC with respect to T0G0, and a significant increasing trend was observed compared to high-grade NMIBC with respect to T0G0 (p = 0.057). ROC curve analysis showed that SUMO1P3 is a promising biomarker for predicting aggressive tumor phenotypes, with an area under the curve (AUC) of 0.848 (low- vs high-grade NMIBC) and 0.878 (low-grade NMIBC vs MIBC) (**Figure 3A**). UCA1 had similar performance in discriminating low- vs high-grade NMIBC (AUC 0.855) and lower performance in discriminating low-grade NMIBC vs MIBC (AUC 0.763) (**Figure 3B**). Considering that combinations of tumor markers can improve diagnostic accuracy, a multivariate logistic regression model was used in the validation set to establish the selected lncRNA panel. The AUC of the panel were 0.872 (low- vs high-grade NMIBC) and 0.875 (low-grade NMIBC vs MIBC), indicating that the combination of the two lncRNAs enhanced the prognostic power with respect to the single lncRNAs, especially in the case of UCA1 alone (**Figure 3C**). SUMO1P3 maintains a comparable or slightly lower sensitivity/specificity, suggesting that it has a robust discrimination ability, which can be considered to establish a prognostic panel.

**Figure 4:**
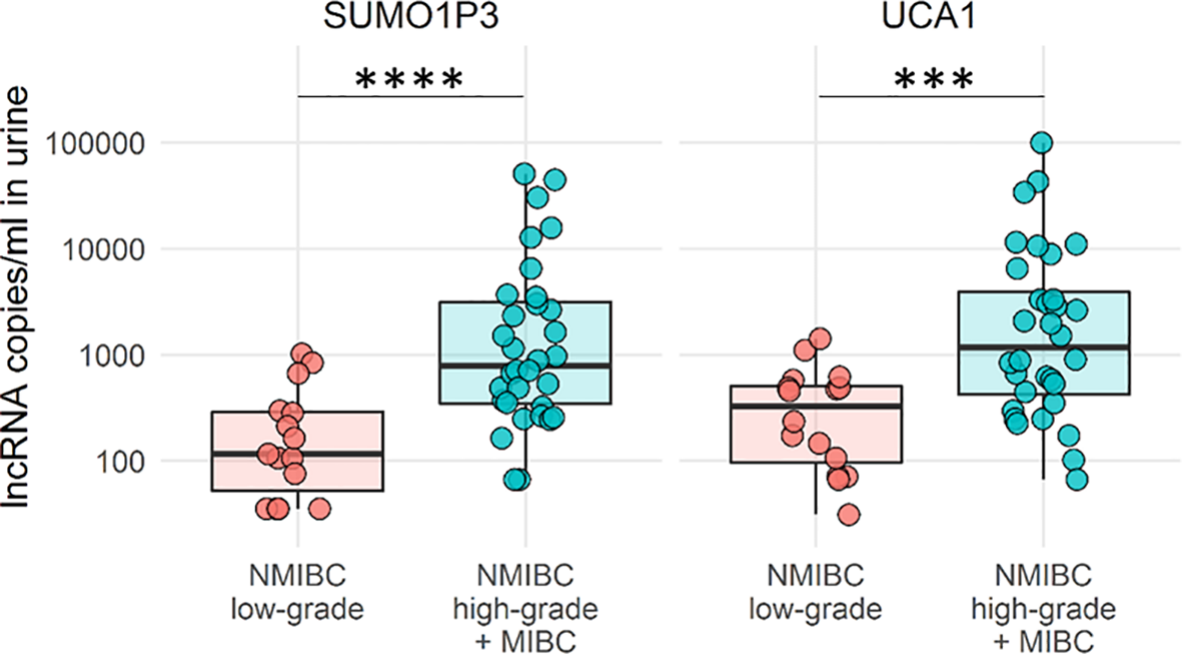
Boxplots of SUMO1P3 and UCA1 levels in low-grade NMBIC samples (red) vs high-grade NMIBC and MIBC samples pooled together (light blue). The Mann–Whitney test was used to compare the levels of each lncRNA between groups (****p-value <0.0001; ***p- value <0.001).

To better empathize with the discrimination power of our lncRNA to delineate tumor progression, high-grade NMIBC and MIBC were pooled and compared to low-grade NMIBC. High-grade NMIBC is an aggressive tumor type with an undifferentiated phenotype that often evolves into MIBC and can be surgically treated in a similar manner (**Figure 4**). The combination of these two lncRNAs strengthens the ability of lncRNAs to discriminate between low-grade versus high-grade invasive tumors.

The overall results demonstrated that SUMO1P3 is a highly specific biomarker for bladder cancer in non-invasive liquid biopsy analysis and can discriminate low-grade from high-grade NMIBC and MIBC, which represent aggressive and invasive phenotypes of the tumor. The SUMO1P3 discrimination power was superior to that of the well-known UCA1, and a combination of the two lncRNAs could be proposed as part of a small biomarker panel.

### The SUMO1P3-associated miR-320a is reduced in high-grade NMIBC and MIBC

**Figure 5:**
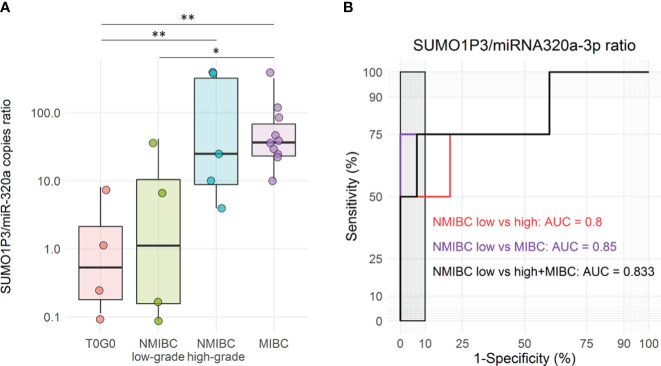
**(A)** Boxplots of SUMO1P3/miR-320a levels ratio in a subgroup of urine samples from bladder cancer patients and controls, included in the validation cohort (n = 4 T0G0, n = 4 NMIBC low-grade, n = 5 NMIBC high-grade, and n = 10 MIBC). The ANOVA test with the Tukey HSD *post-hoc* analysis was used to determine significant differences between groups (**p-value <0.01, *p-value <0.05). **(B)** Receiver Operating Characteristic (ROC) curves for SUMO1P3/miR-320a levels ratio in low-grade NMBIC vs high-grade NMIBC samples (red), low-grade NMBIC vs MIBC samples (violet), and low-grade NMBIC vs high-grade NMIBC and MIBC samples pooled together (black). The corresponding ROC-AUCs are reported on the graph. The gray rectangle highlights the area in which the specificity of the test is >90%.

Since SUMO1P3 has been demonstrated to bind to miR-320a in breast cancer (11) and hepatocellular carcinoma (21) facilitating tumor progression, we investigated the presence of miR-320a in the urine of bladder cancer patients and controls. Using ddPCR, we quantified miR-320a levels in a subgroup of urine samples with higher concentrations, where a part of the sample was still available. Since miR-320a levels were supposed to be lower in tumor samples than in healthy controls and inversely correlated with SUMO1P3 levels, we determined the capacity of the SUMO1P3/miR-320a ratio in discriminating cases from controls (**Figure 5**).

Although the number of samples was lower compared to previous analyses, the combination of SUMO1P3 and miR-320a showed a significant difference between controls/low-grade NMIBC and high-grade NMIBC/MIBC (**Figure 5A**). ROC curves analysis showed that SUMO1P3/miR320a were significant predictors of progression outcomes with an AUC = 0.8 (low- vs high-grade NMIBC) and 0.85 (low-grade NMIBC vs MIBC), as well as when high-grade NMIBC and MIBC were pooled (AUC = 0.833) (**Figure 5B**).

## Discussion

Urine is a reliable source of biomarkers and has a special role in urological malignancies, as many of the substances directly secreted from the tumor mass or contained in tumor extracellular vesicles (EVs) are likely to drain directly into the urinary tract, and exfoliated cancer cells and cellular debris can be found in the first morning urine (22). The advantages of urine as a liquid biopsy include collection with a non-invasive procedure, availability of large and regular quantities, suitability for longitudinal follow-up, detection of intra-tumor heterogeneity, and limitations represented by the effect of hydration status and medications, RNA extraction, enrichment and measurement, and variability in assay protocols/sample handling due to the lack of standardization (22). These drawbacks can be overcome using standardized preanalytical procedures and quantitative techniques. Here, we show that the amount of lncRNA SUMO1P3 is enhanced in the urine of patients with high-grade NMIBC and MIBC compared to healthy controls and low-grade NMIBC. In addition, we demonstrated that SUMO1P3 could discriminate unrelated diseases, such as BPH, from aggressive bladder cancer. SUMO1P3 is a pseudogene that was found to be upregulated in bladder cancer, and its expression was positively correlated with greater histological grade (13) as well as in other tumor tissues, such as gastric (9), colon (10), breast (11), and liver (12) cancers. We proved for the first time that SUMO1P3 can be detected in urine and that its presence is correlated with bladder cancer grade and stage, resulting in a promising, non-invasive biomarker for tumor progression. Its performance is comparable or even better than that of UCA1, another lncRNA that has long been established to be involved in bladder cancer progression (23). It is noteworthy that the combination of the two constitutes a small panel with good power (AUC 0.872) to discriminate low-grade from high-grade NMIBC and can be useful in specific clinical conditions, such as the monitoring of patients who are subjected to transurethral resection of bladder tumor (TURBT) and are under surveillance, but also as primary screening to detect high-grade NMIBC or in cases of inconclusive/suspicious urinary cytology.

Other urinary lncRNAs have been proposed alone or in combination to determine their diagnostic utility in bladder cancer, such as TERC (24), the twosome MIR205HG-GAS5 (25), and the 3-lncRNA panels MALAT1, PCAT-1, and SPRY4IT1 (26), which exhibited good differentiating ability between healthy and diseased subjects. The lncRNA TERC levels were monitored in urine exosomes, showing an AUC of 0.836, while that of the reference protein, FDA-approved, NMP-22 was 0.696 (the AUC of combined indicators reached 0.861) (24). The combined panel of two exosomal lncRNAs, MIR205HG and GAS5, for diagnostic purposes displayed an AUC of 0.842, which could be integrated with other contributions, including mRNAs (25). A panel consisting of the three lncRNAs MALAT1, PCAT-1, and SPRY4-IT1 was established for BC diagnosis, showing an AUC of 0.854 (26). Another study investigated the performance of lncRNAs in urine fractions for BC diagnosis, and a higher combined diagnostic result was achieved by RMRP, UCA1, and MALAT1 in urinary exosomes (AUC 0.875) (27), confirming that lncRNAs may represent an appropriate group of biomarkers for BC detection. GAS5 together with uc004cox.4 provided high diagnostic accuracy of BC with an AUC of 0.885 (28). The expression of the five mRNA targets ABL1, CRH, IGF2, UPK1B, and ANXA10, frequently over-expressed in BC have been detected in voided urine through the Xpert Bladder Cancer Monitor assay, reaching an AUC of 0.87 (29). From a prognostic point of view, a global miRNA expression profiling analysis identified in the urine pellet a two miRNA signature composed of miR-92a and miR-125b, which exhibited an AUC of 0.83 (30).

Recently, SUMO1P3 has been associated with miR-320a in the development of breast cancer (11) and hepatocellular carcinoma (21). Notably, miR-320a was found to be downregulated in bladder cancer cells, and it was proposed to function as a tumor suppressor by targeting the integrin subunit beta 3 gene (ITGB3) and inhibiting cell invasion (31). We found reduced miR-320a in the urine of bladder cancer patients with high-grade NMIBC and MIBC, correlating with a greater amount of SUMO1P3. The SUMO1P3/miR-320a ratio combines both sides of the same coin and might be a valuable instrument with respect to the single components. We hypothesized that the tumor-promoting effects of SUMO1P3 in bladder cancer may be partially mediated by miR-320a negative regulation, as occurs in breast cancer (11), and that the molecular mechanism consists of sequestering miRNA by sponging through miRNA binding sites, already identified in the SUMO1P3 sequence (11, 21).

In conclusion, we have shown that SUMO1P3 is increased in the urine of patients with high-grade NMIBC and MIBC and that it is a good candidate for monitoring bladder cancer progression. It has good discrimination power if used alone or in combination with the other lncRNA UCA1 or with its interactor miRNA320a.

## Data availability statement

The original contributions presented in the study are included in the article/**Supplementary Material**. Further inquiries can be directed to the corresponding author.

## Ethics statement

The studies involving humans were approved by the Institutional Ethics Committee of San Raffaele Hospital (protocol code URINEBIOMAR, date of approval: 04/08/2016). The studies were conducted in accordance with the local legislation and institutional requirements. The participants provided their written informed consent to participate in this study. Written informed consent was obtained from the individual(s) for the publication of any potentially identifiable images or data included in this article.

## Author contributions

SG: Formal analysis, Investigation, Writing – review & editing, Data curation, Methodology, Validation. AB: Formal analysis, Writing – review & editing. GC: Formal analysis, Writing – review & editing. CS: Formal analysis, Writing – review & editing. FT: Formal analysis, Writing – review & editing. GV: Formal analysis, Writing – review & editing. IM: Software, Writing – review & editing. MG: Formal analysis, Writing – review & editing. RP: Resources, Writing – review & editing. FM: Resources, Writing – review & editing. AS: Resources, Writing – review & editing. RV: Conceptualization, Formal analysis, Funding acquisition, Investigation, Project administration, Supervision, Writing – original draft, Writing – review & editing.
